# Molecular Time-Course and the Metabolic Basis of Entry into Dauer in *Caenorhabditis elegans*

**DOI:** 10.1371/journal.pone.0004162

**Published:** 2009-01-08

**Authors:** Pan-Young Jeong, Min-Seok Kwon, Hyoe-Jin Joo, Young-Ki Paik

**Affiliations:** Department of Biochemistry, College of Life Sciences and Biotechnology, Yonsei Proteome Research Center and Biomedical Proteome Research Center, Yonsei University, Seoul, Korea; Texas A&M University, United States of America

## Abstract

When *Caenorhabditis elegans* senses dauer pheromone (daumone), signaling inadequate growth conditions, it enters the dauer state, which is capable of long-term survival. However, the molecular pathway of dauer entry in *C. elegans* has remained elusive. To systematically monitor changes in gene expression in dauer paths, we used a DNA microarray containing 22,625 gene probes corresponding to 22,150 unique genes from *C. elegans*. We employed two different paths: direct exposure to daumone (Path 1) and normal growth media plus liquid culture (Path 2). Our data reveal that entry into dauer is accomplished through the multi-step process, which appears to be compartmentalized in time and according to metabolic flux. That is, a time-course of dauer entry in Path 1 shows that dauer larvae formation begins at post-embryonic stage S4 (48 h) and is complete at S6 (72 h). Our results also suggest the presence of a unique adaptive metabolic control mechanism that requires both stage-specific expression of specific genes and tight regulation of different modes of fuel metabolite utilization to sustain the energy balance in the context of prolonged survival under adverse growth conditions. It is apparent that worms entering dauer stage may rely heavily on carbohydrate-based energy reserves, whereas dauer larvae utilize fat or glyoxylate cycle-based energy sources. We created a comprehensive web-based dauer metabolic database for *C. elegans* (www.DauerDB.org) that makes it possible to search any gene and compare its relative expression at a specific stage, or evaluate overall patterns of gene expression in both paths. This database can be accessed by the research community and could be widely applicable to other related nematodes as a molecular atlas.

## Introduction

The nematode *Caenorhabditis elegans* is a rapidly growing worm that completes its entire life cycle—from egg to adult—in only 3.5 days in the presence of abundant food (at 20°C) [1]. When developing *C. elegans* larvae sense the dauer-inducing pheromone [2], or daumone [3], they enter into a physiologically specialized condition—the dauer state [4]. Daumone [3] or its analogues [5] signal to the worms that local conditions are unfavorable for growth (e.g., inadequate food supply or overcrowding), and induce entry into diapause, a process called dauer entry. Dauers possess a very thin body, contain large amounts of fat, do not age, and are able to endure adverse conditions [6]. They can survive for several months [7] in this condition, and are capable of re-entering their life cycle when conditions are again favorable for growth [1], [2]. However, the duration of the dauer larval stage appears to have important developmental and reproductive consequences [8]. The sensory response to daumone appears to be mediated by amphid neurons [9] via a process in which the G protein subunit, GPA-3, appears to serve a gating function [10]. It has recently been shown that fluorescent daumone analogues are also transported to amphid neurons and induce the dauer state [11].

Several previous reports have described genome-wide molecular profiling in *C. elegans*. These studies have addressed TGF-β-dependent transcriptional changes, but only in the dauer stage [12]; monitored differences in expression in dauer larvae in a *daf-2* mutant [13]; followed changes in gene expression that accompany the dauer recovery process [14]; and analyzed dauer larvae of wild-type N2 worms using serial analysis of gene expression (SAGE) [15]. However, there have been no reports in which real-time monitoring has been employed to characterize the worm-wide time-course of gene expression during the entire dauer process. Nor have there been any attempts to construct a systematic database using well-controlled dauer-inducing conditions (e.g., the presence of pure daumone as the single dauer-inducing signal). Because the dauer/non-dauer decision is critical for the survival of *C. elegans* under harsh environmental conditions [1], identifying and globally dissecting these previously uncharacterized molecular paths, as well as defining metabolic regulation during this developmental transition, are critically important for understanding this *C. elegans* survival strategy. Here we report the previously unexplored molecular landscape of an entire dauer entry process, following these changes across multiple time points along two different paths. Our findings provide a plausible molecular basis for differences between energy utilization in the pre-dauer and the dauer-maintenance state of *C. elegans* in the context of prolonged survival under adverse growth conditions.

## Results

### Establishment of the Molecular Time-Course for Dauer Entry

We initiated our molecular profiling of the dauer entry process by asking two important questions: First, when worms are grown in the presence of a concentration of dauer pheromone that is optimal for dauer induction, is there a defined metabolic timeline from the perception of the daumone signal to appearance of dauer larvae? Second, what is the metabolic basis for the worm's adaptation during the developmental shift to diapause? We addressed these questions systematically, using DNA microarray analysis to quantitatively measure changes in gene expression patterns throughout dauer entry in real time, emphasizing those genes involved in energy-generating metabolic pathways (e.g., fats and carbohydrates).

**Figure 1:**
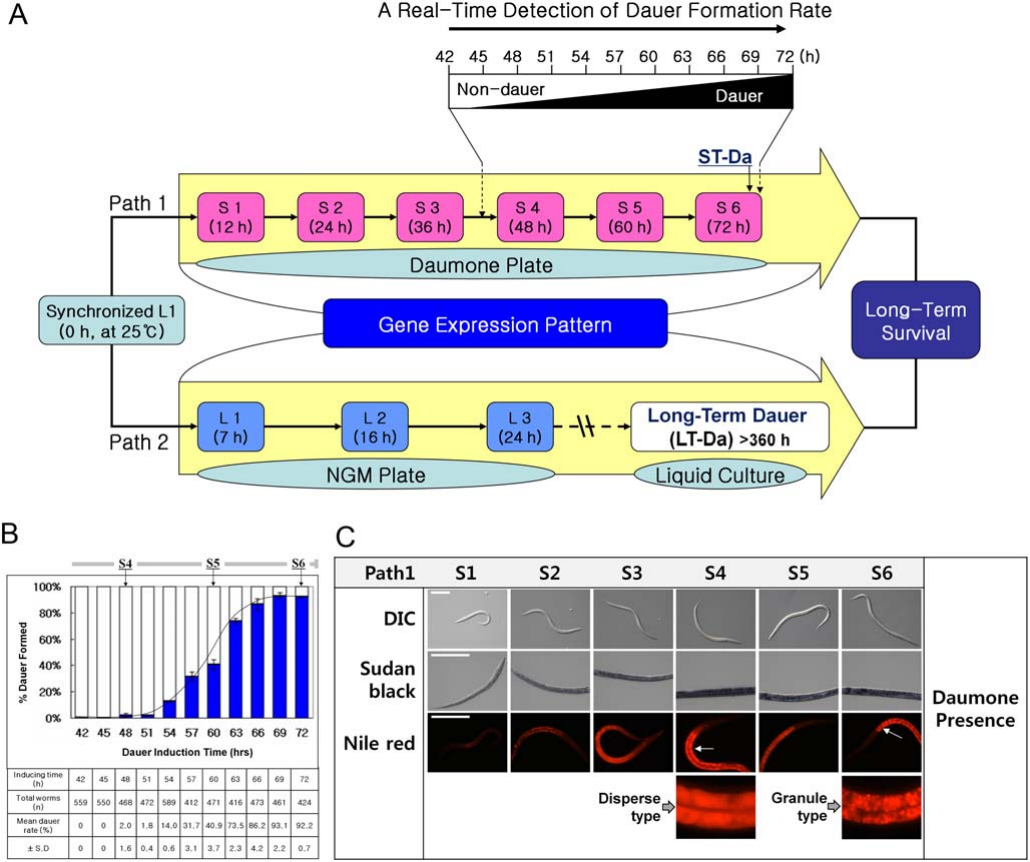
An overview of the systematic approach used to analyze molecular pathways of Dauer entry in *C. elegans*. (A) Short-term dauer larvae were induced by spreading <200 synchronized N2 worms (L1) on daumone plates (4.52 cm^2^) in the presence of dead *E. coli* as described [2] (Path 1, developing pheromone dauer). For long-term dauer induction, worms were grown on an NGM plate (Path 2, developing fed larvae) to the L3 stage at 25°C. Worm samples were then collected at different time points. The short-term dauer larvae (ST-Da) were those collected at S6 (72 h), whereas long-term dauer larvae (LT-Da) were prepared by transferring worms collected at the end of the L3 stage to liquid culture and growing for >360 h, as described (2, 4). (B) Dauer induction rate curve showing quantitative measurement of *C. elegans* dauer formed on daumone plates at each stage. After incubation of worms on a daumone plate (Path 1), the worms showing a dauer phenotype were collected every 3 h between 42 h and 72 h and treated with 1% SDS for 30 min. Surviving worms were counted as authentic dauer larvae. (C) Worms stained at each stage in Path 1 for qualitative measurement of fat accumulation during Dauer entry.

As shown in Figure 1A (top), Path 1, designed for analyzing the entry process that develops in the presence of pheromone, or short-term duaer larvae (ST-Da) formation, permits monitoring of both the number of dauer larvae and the levels of mRNA expression in worms grown in the presence of the optimal concentration of daumone [3], without interference from other factors (e.g., mutations, high temperature, limited food supply or population density). Path 2, designed for analyzing developing fed larvae, allows us to monitor transcriptomic changes that occur during the regular life cycle of *C. elegans* as well as those associated with long-term dauer larvae (LT-Da) (Figure 1A, bottom). In this path, stage-specific expression data (L1, L2 and L3) is generated from fully fed worms grown on plates without daumone, and thus serves as a control dataset for Path 1 data obtained from worms exposed to daumone. In general, ST-Da are formed under daumone-only conditions, whereas LT-Da are induced by multiple factors associated with unfavorable growth condition, including overcrowding, food deprivation and dauer pheromone. Thus, transcriptomic analysis of these two different conditions provides complementary information that should prove helpful in comparing dauer-induction and dauer-maintenance gene expression patterns in dauer larvae facing different durations of diapause. Dauer induction as a function of time for Path 1 is presented in Figure 1, which shows both an individual, variable rate of entry into the dauer state and a general dispersion of response rate/metabolism. From this process, we observed that dauer larvae formation begins at post-embryonic stage S4 (∼2.0% dauers; *n* = 468) after a certain preparation period (48 h), and is complete at 69 h (≥93.1%±2.2% dauers; *n* = 461). This dauer induction rate curve is also further corroborated by the morphological changes (e.g., thin body and resistance to 1% SDS) and differential fat accumulation (Figure 1C) characteristics of dauer larvae. Nile-Red staining revealed that fat accumulation starts at S2 (24 h after exposure to daumone) and peaks at S6 (ST-Da) when fat deposition appears granular, indicating a qualitative change in fat metabolism during dauer entry (Figure 1C).

### Construction and Application of a Versatile Dauer Metabolic Database

**Figure 2:**
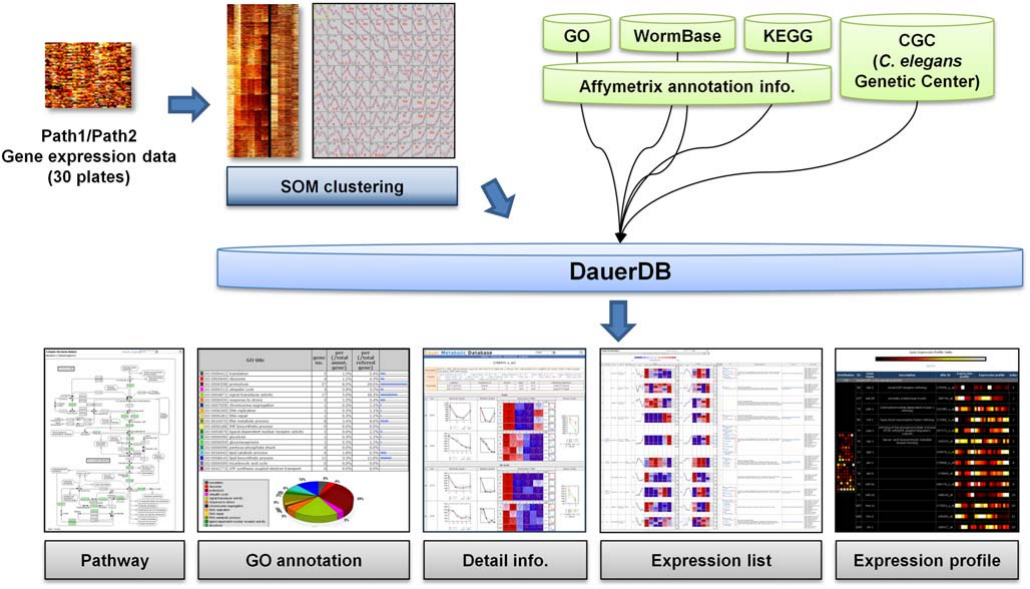
Structure of DauerDB. *C. elegans* expression data from a total of 30 microarray plates obtained from Path 1 and Path 2 analyses were clustered by SOM and annotated based on the Affymetrix gene DB. Annotated information for 22,626 genes and information obtained from GO, WormBase, KEGG and CGC were integrated into DauerDB (www.dauerdb.org).

To systematically monitor changes in the levels of mRNA transcribed from specific genes during dauer entry, we used a DNA microarray containing 22,625 gene probes corresponding to 22,150 unique genes from *C. elegans* (http://www.affymetrix.com/index.affx). The relative expression of these genes was monitored at six time points, S1, S2, S3, S4, S5, S6 (ST-Da), in Path 1, and four time points, L1, L2, L3 plus LT-Da, in Path 2. This information was compiled into a web-based DNA microarray database (DB) for dauer entry (DauerDB), which was constructed as depicted in Supplemental Figure 1S. Using DauerDB (www.dauerdb.org), it is possible to search for any gene (or genes) and compare its (their) relative expression at a specific stage or evaluate overall patterns of gene expression in both Paths (Figure 2). In addition, this portal provides access to the web-based NetAffx-supported DB, WormBase (http://www.wormbase.org/), and the KEGG pathway DB (http://www.genome.jp/kegg/), both of which have annotation information. Based on the expression profiles of genes as deposited in DauerDB, a one-dimensional hierarchical clustergram was created using the self-organizing map (SOM) clustering method [16], which transformed numerical values from a total of 30 arrays (three arrays per growth time point) into a color scale [16] (Figure S2). The different color intensities in the ten sample sets, S1, S2, S3, S4, S5, S6 (ST-Da) and L1, L2, L3 plus LT-Da, reflect the average expression level per sample set (Figure S2). To assess how worms prepared metabolically for unfavorable environmental conditions, we classified microarray data at the appropriate time points and growth stages by signal intensity. On the basis of this classification procedure, twelve types of expression patterns can be discerned that generally fall into one of three main classes: (i) stage-specific expression, for those genes displaying peak expression at a certain stage followed by a decrease in message level (e.g., S1, S2, S3, S4, S5-type expression); (ii) dauer-specific expression, for those genes displaying a peak in expression at the dauer stage (e.g., ST-Da and LT-Da type); and (iii) an irregular response (IR) (Figure S2).

### Altered Expression of Genes Involved in Aging-Related Signaling during Dauer Entry

**Figure 3:**
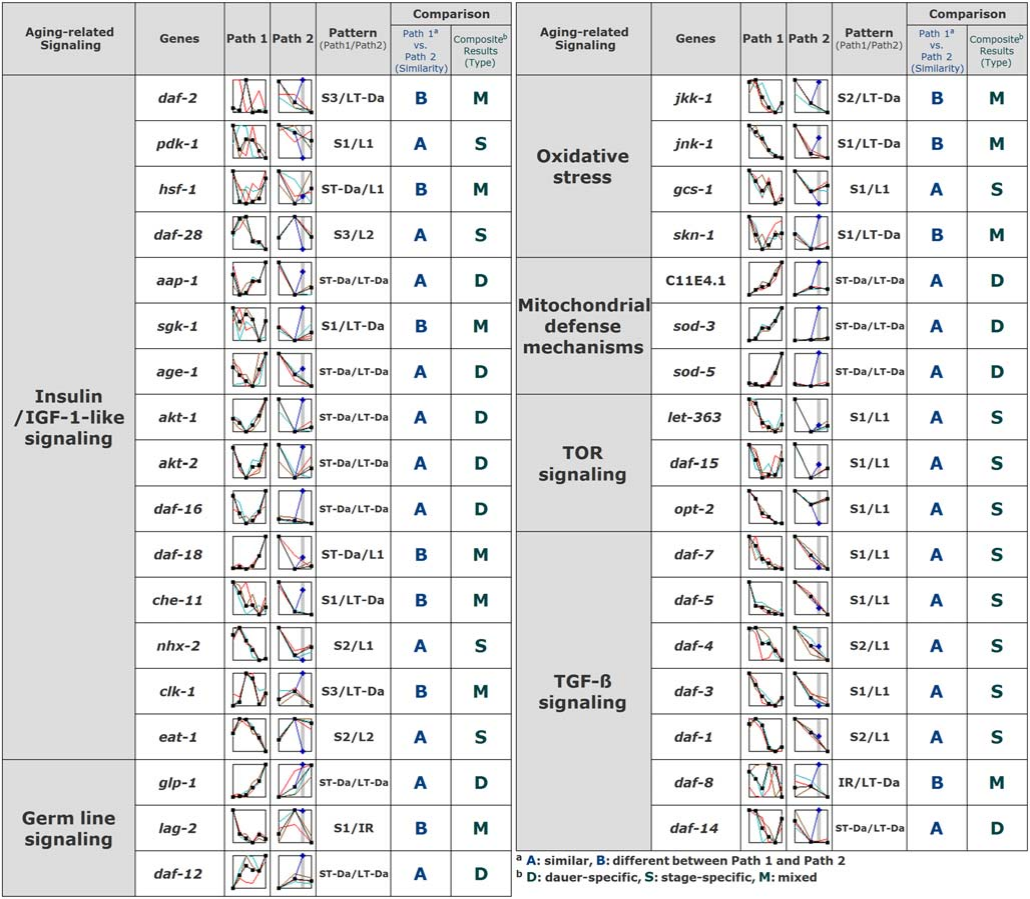
Differential expression patterns of genes involved in aging-related metabolic pathways during dauer entry in both Path 1 and Path 2 developmental paradigms. Shown here are the representative genes clustered as aging genes and their relative expression at different time points (from triplicate samples).

To investigate how genes involved in the aging signaling pathway respond to each time-course of dauer entry, we examined changes in the transcription patterns of genes selected on the basis of the proposed classification of aging-related genes [18]. The transcriptional changes in representative genes from each group were further verified by quantitative reverse transcription-polymerase chain reaction (qRT-PCR) (Figure S3). The identified genes include those from the insulin/IGF-1 like signaling (IlS) [19]–[25], JNK, TOR and TGF-β pathways, as well as genes involved in oxidative stress, germ-line development and mitochondrial defense mechanisms. In general, most genes in the TGF-β and TOR signaling pathways were suppressed, whereas a preponderance of those typical of mitochondrial defense (3/3), oxidative stress (3/4), germ-line signaling (2/3) and IlS (5/15) were highly induced in a dauer-specific manner, regardless of paths (Figure 3). This result suggests that a metabolic shift at dauer stage appears to drive a concerted modulation in aging-related metabolic genes that leads to prolonged survival [26]–[28].

### Cooperative Gene Regulation in Fat Metabolism during and after Dauer Entry

Since the most remarkable feature of dauer larvae is their dramatic accumulation of body fats (Figure 1C), triacylglycerol (TG) might be critical for survival during or after dauer entry. To investigate whether there is a molecular mechanism for fine control of lipid metabolism during or after dauer entry, we examined the expression of representative fat genes.

**Figure 4:**
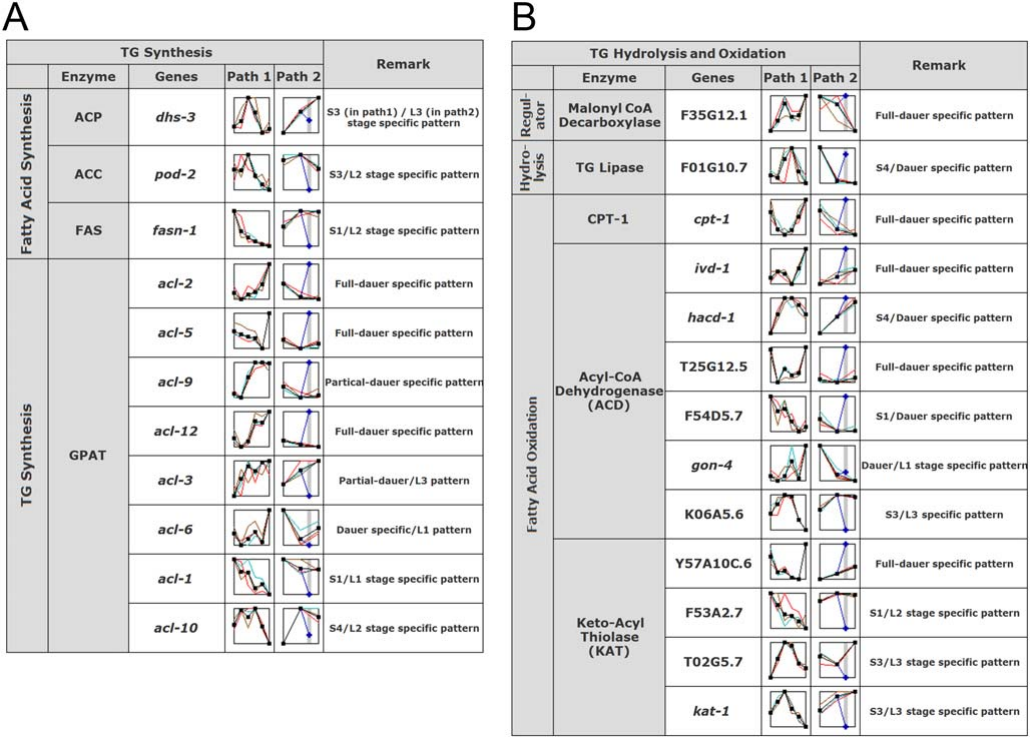
Differential expression patterns of genes involved in fatty-acid metabolism during dauer entry. The expression patterns for representative genes involved in biosynthesis (A) and hydrolysis and oxidation of triacylglycerol (B) are shown. The expression graph in each path is the same as that in Figure 3B. Full and partial dauer-specific induction denotes gene induction in both dauer states and in only one dauer state, respectively.

First, we monitored changes in the expression of TG synthesis genes in both dauer states (Figure 4A). The expression of most fat genes examined was also verified by qRT-PCR (Figure S4). As anticipated, the genes for 3 acyl carrier protein (ACP) and acetyl CoA carboxylase (ACC), which act at the early stage of FA synthesis, shared a similar stage-specific expression pattern in both paths (Figure 4A; see also Figure S4). The expression of the fatty acid synthase (FAS) gene was highest at S1 (12 h) in Path 1, but peaked at L2 (16 h) in Path 2. In Path 1, this fatty acid (FA) pool may result from both *de novo* FA biosynthesis and from the absorption of FA from ingested foods before worms enter the dauer state at S4 (Figure 1C). After FA biosynthesis, worms would convert FA to TG using glycerol-3-phosphate (predicted to be produced immediately after S3) as a substrate for glycerol-3-phosphate acyltransferase (GPAT) in both paths [24]. In fact, TGs function as long-term energy storage molecules and are cleaved to FA as needed in dauers [25]. As shown in Figure 4A, the pattern of GPAT expression varied depending upon the experimental path. In both paths, six *acl* genes showed some degree of dauer-specific induction, with *acl-2*, *-5*, *-9*, *-12* exhibiting full dauer-specificity, and *acl-3* and *-6* exhibiting partial dauer-specificity. (See also legend to Figure 4 for description of full and partial dauer-specific expression.) In contrast, *acl-1* decreased gradually throughout aging, and *acl-10* decreased specifically in both dauer stages. The fact that the majority of predicted GPATs (6/8) in *C. elegans* were highly induced in both dauer states suggests that *de novo* TG synthesis might also occur in both dauer states, as noted above (see Figure 1C). Data from qRT-PCR were in good agreement with DNA microarray data (Path 1), confirming the validity of the overall pattern of expression of genes involved in lipid metabolism (Figure S4). Furthermore, knock-down of GPAT homologs using RNA interference (RNAi) profoundly influenced the extent of fat accumulation in the background of N2, *daf-2*, *daf-7* and *nhr-49* strains (data not shown).

Second, we monitored changes in FA-oxidation genes along both paths. Note that oxidation of FA in mitochondria is a major means of producing metabolic energy that may be crucial for long-term survival of worms during or after the dauer state. During normal development, the hydrolysis of TG (the first step in the release of stored FA for energy production) must be coordinately regulated with TG synthesis to ensure a balance between adequate energy stores and utilization. Oxidation of FA begins by activation to a fatty acyl-CoA in the endoplasmic reticulum (ER) or outer mitochondrial membrane; thereafter, the acyl-group is transferred to carnitine in a reaction catalyzed by carnitine palmitoyl transferase (CPT)-1 [29]. For FA oxidation to occur, the level of malonyl-CoA, which negatively regulates acyl-CoA transfer to carnitine during transit to the mitochondria, must fall; this is probably accomplished by activation of malonyl-CoA decarboxylase (MCD). Consistent with this interpretation, we found that MCD was induced in a dauer-specific manner in both paths (Figure 4B). Subsequently, TG lipase (F01G10.7) was induced at S3 in Path 1 and at LT-Da in Path 2, indicating that TG hydrolysis is differentially initiated in short-term and long-term dauers. Next, we scrutinized two key classes of enzymes, acyl-CoA dehydrogenase (ACD) and β-ketothiolase (KAT), which catalyze the first and final reaction steps in β-oxidation, respectively. Collectively, these genes (6 of 11 examined) showed a full or partial dauer-specific pattern; thus, FA oxidation is most likely highly active primarily in both dauer stages. In addition to the energy provided by utilizing TG, proteolysis might also provide raw material for carbohydrate-based energy molecules. These would be derived from the pool of pyrimidine and purine precursors created by proteolytic processing for use in DNA and RNA synthesis at S6 (ST-Da) (Figure S5). Thus, the presence of a coordinated regulatory mechanism for fat metabolism and dauer formation likely represents a key strategy for the long-term survival of worms under various harsh environments.

## Discussion

**Figure 5:**
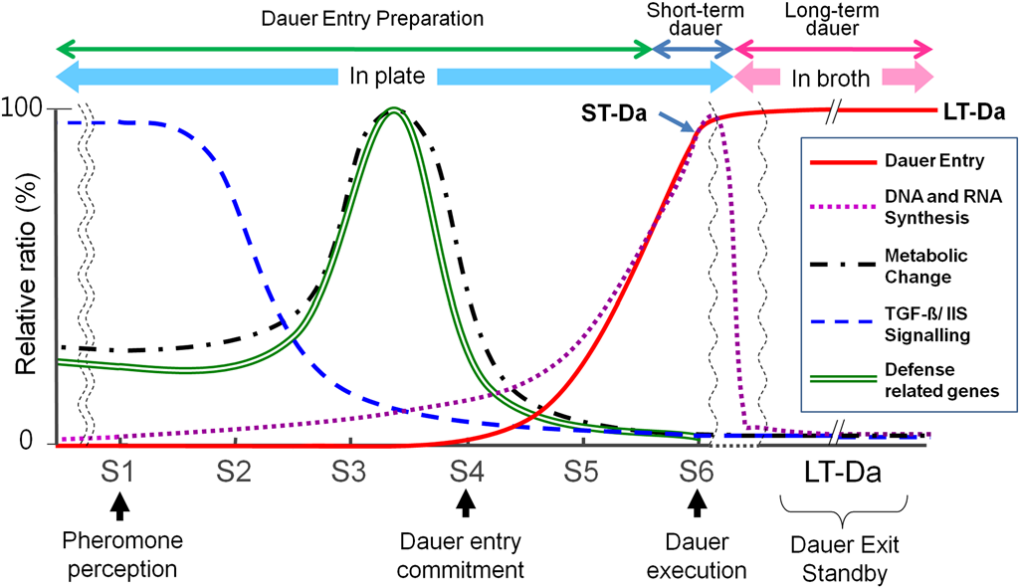
Prediction of the global metabolic landscape during entry into the dauer state. The x-axis indicates the approximate timeline of development for worms grown on daumone plates and in broth culture (LT-Da), whereas the y-axis indicates the level of gene expression (in arbitrary units) of each pathway deduced from DauerDB. Composite information relating trend of dauer entry, DNA and RNA synthesis, major energy metabolic pathways, TGF-β/IlS signaling and defense-related genes are displayed as function of time.

This report marks the first analysis of the molecular landscape of dauer entry in the wild-type N2 strain grown under two different experimental conditions (Figure 1). With respect to dauer entry on a daumone plate (Path 1), two points should be highlighted (Figure 1B). First, there might be a minimum preparation period (≥48 h here) early in the process, possibly within the first hours after hatching, during which worms sense daumone and respond to it by a gradual reprogramming of growth that culminates in dauer formation. However, we do not exclude the possibility that the dauer induction rate curve presented in Figure 1B also reflects, in part, detection of an asynchronous response in L1 animals. We attempted to minimize asynchronous responses among individuals by keeping the ratio of unhatched eggs to synchronized L1 below 1/1000. Although we don't know what happened during this 0-to-48-h preparation period, we clearly saw dauer larvae with closed mouths and dauer cuticles starting at S4 (48 h). Microscopic analysis (Fig. 1C) also supports the conclusion that ingested Nile-red stains were condensed in fats or incorporated into TG before worms entered the dauer stage; these changes may be related to cuticle formation. We therefore designated S4 as the ‘dauer entry commitment point’, marking the beginning of a gradual developmental arrest and morphological changes (Figure 5). Second, different metabolic pathways exhibited different fluxes with increasing duration of daumone exposure, as predicted by changes in corresponding pathway genes throughout the course of dauer formation (i.e., ST-Da). This is also supported by the fact that synthesis of FA peaked between 12 h (FAS) and 36 h (ACP and ACC). In general, metabolic activity and expression of defense-related genes peaked at S4 (Figure 5), whereas DNA/RNA synthesis and dauer induction processes appeared to run in parallel. The TGF-β signal was continuously down-regulated as the dauer entry process progressed until the dauer execution point at 72 h (S6, ST-Da).

Previous genome-wide surveys directed toward increasing our understanding of the dauer stage have focused on transcriptional alteration in the context of dauer recovery [14] or on dauer formation in various mutants (e.g., *daf-7*, *daf-2*, *fer-15*, *spe-9*, *emb-27* and *daf-16*) [11], [30]–[32], as opposed to dauer entry in wild-type N2. Some apparently fundamental differences in the results obtained in these studies may be due to differences in study goals, as reflected in the specific experimental designs and conditions used. In the context of dauer metabolic regulation, our experiments employing wild-type N2 may come closest to replicating the real-world conditions that initiate longevity mechanisms in N2 worms. For the series of studies in Path 2, the inclusion of both L1, L2, L3 and LT-Da data was necessary because there had been no previous reports comparing gene expression profiles between non-dauer and LT-Da at the time these experiments were initiated. Our results can be used as reference source for profiling of both genes involved in LT-Da (and maintenance of dauer) and those involved in development from stage L1 to L3. In these experiments, we made repeated attempts to induce LT-Da on NGM plates in order to reduce the heterogeneity of the LT-Da population, but found that worms frequently crawled out of the plates and eventually died. To minimize population heterogeneity, we cultured a high-density population of synchronized L1 stage in the presence of excess food at 25°C. After 7 days, when most of the nutrients had been depleted, we found that 80% of the population entered the dauer stage. These worms were cultured for an additional 8 days without food, after which dauer worms were selected by 1% SDS treatment. Despite the possible presence of co-existing progeny, the LT-Da population obtained in this manner was likely to be highly homogenous.

In worms that reach the dauer stage, proteins are regenerated in order to minimize energy consumption; TOR kinase signaling, which responds to amino acid level, is important in establishing this relationship between nutritional level and dauer stage [33], [34]. At higher amino acid levels, TOR signaling up-regulates translation through activation of ribosomal S6 kinase (S6K), while at lower amino acid levels, it promotes autophagy, leading to degradation and turnover of proteins. IIS also prevents formation of the developmentally arrested dauer larva in *C. elegans* [1], suggesting crosstalk between IIS and TOR signaling. If TOR and IIS act together, reduced TOR activity would be predicted to result in constitutive dauer larvae formation, since mutations in the *C. elegans* TOR gene (*let-363*) have been shown to cause mid-larval arrest [35], [36]. Our studies on the effects of sustained suppression of all three genes encoding TOR kinases (*let-363* [35], [37], *opt-2* (oligopeptide transporter) [38] and *daf-15* [36]) from the early stages (S1, L1) (Figure 3) suggest the presence of a concerted regulatory mechanism that keeps the fuel-utilization system in check in preparation for dauer stage (see below). In Path 1, once worms in the pre-dauer state perceive daumone, amino acids produced from continuing proteolysis throughout dauer entry (S1, S2, S3, S4, S5, S6, ST-Da)(Figure S5), or possibly via autophagy [39], might provide a certain measure of metabolic energy through gluconeogenesis [40], allowing fat-based fuels to be conserved for survival in the dauer state. Such a role for autophagy in *C. elegans* dauer development and life-span extension has been suggested based on studies showing that knockdown of *bec-1* expression shortens the lifespan of the *daf-2 (e1370)* mutant *C. elegans* [39]. In our experiment, genes involved in autophagy (e.g., *bec-1* and *lgg-3*) were highly induced in a dauer-specific manner in LT-Da (Path 2) (www.dauerdb.org), confirming that protein degradation through autophagy provides an alternate metabolic pool (i.e., amino acids) for survival during the dauer state [39], [41], [42]. This is also demonstrated by the fact that most UDP-glucuronosyl transferase (*ugt*) genes (e.g., *ugt-11*, *-14*, *-20*; see www.dauerdb.org) showed dauer-specific induction, suggesting a role in removing metabolic wastes that might otherwise accumulate due to continued proteolysis during dauer entry.

Since fat oxidation is regarded as a major fuel-utilization pathway during the dauer stage, it is reasonable to presume that expression of members of the *fat* gene family (Figure 4A and B) would be closely coordinated to satisfy cellular demands for metabolic building blocks, either for regular developmental process during the pre-duaer stage or for energy production during long-term dauer survival [43]. However, we found that TG synthesis was delayed and peaked at the dauer stage, a time when TG was thought to serve as an energy source; moreover, TG hydrolysis was shown to be most active at the S4 stage (48 h point). Why this would be the case is not clear. The level of TG lipase (F01G10.7) expression first decreased below that of genes involved in FA or TG synthesis beginning at S4 (Figure 4B). Furthermore, the expression of a family of TG lipase genes (F45E6.4, C06G6.3, F31B12.1A and W02B12.1) continuously decreased throughout Path 1 (DauerDB). To reconcile these seeming contradictions, we might speculate that carbohydrate-based fuels and residual TGs must first be consumed before nascent TG synthesis can be initiated, beginning at the pre-dauer stage and peaking at the dauer stage. The energy released by drawing on these pre-existing fuel sources might also be used for the synthesis and/or modification of cuticles and other defense proteins in preparation for the dauer stage and the expectation of a slowdown in metabolic fuel utilization. At the same time, an increase in the expression of TG synthesis genes at S6 may reflect a need for worms to build up TG reserves in the body for long-term survival (Figure 1C). Using Sudan black staining, we observed that there was no significant decrease in fat accumulation in either LT-Da or ST-Da animals. Consistent with previous studies by Riddle and colleagues and others [44], [45], we found using a homologue display incorporating the dauer metabolic pathway into the KEGG pathway map (DauerDB) that genes involved in ATP synthesis were decreased.

Collectively, our data suggest that pre-dauer larvae rely heavily on carbohydrate-based energy reserves whereas dauer larvae utilize a fat or glyoxylate cycle-based energy source, as suggested previously [14], [25]. Analysis of DauerDB also supports the conclusion that three carbohydrate-based energy pathways—glycolysis, the pentose phosphate shunt and gluconeogenesis—are highly active at S3 during dauer entry (Path 1) (Figure S4). In dauer states (dauer-specific induction), FA oxidation (Figure 4B) and the glyoxylate cycle were active. In addition to serving as an energy supply, these pathways would be used for the maintenance of the defense enzyme system, which leads to the continuous synthesis of mRNA for dauer-specific genes [1]. Thus, *C. elegans* appears to employ a unique strategy to determine which type of diapause will most likely ensure survival upon exposure to different unfavorable environments.

### Future Prospects and Conclusions

Our studies and the companion comprehensive DauerDB provide molecular insights into the key regulatory mechanisms associated with dauer entry *in vivo* and suggest strategic countermeasures for developing novel anti-nematode compounds against many plant-parasitic nematodes (e.g., the pine wood nematode and soybean cyst nematode) under various harsh environments. For instance, the pinewood nematode, a destructive pest of pines [43] that has caused serious damage in forest ecosystems worldwide, develops into a dispersal form similar to dauer when grown in the insufficient food source, *Botrytis cinerea* (Oh et al., unpublished data). These dauer-like larvae are often present in wilted trees in cold seasons; once they invade the callow *Monochamus altanatus*, however, they can remain in the tracheae of the tree [47]. If the fat-mobilizing system of these worms can be interrupted during dauer entry using a chemical agent, perhaps this nematode can be eradicated. Dauer entry appears to be compartmentalized in time and according to metabolic flux (Figure 5), suggesting that the efficient genome-wide coordination among genes involved in discrete steps (e.g., pheromone reception, dauer entry commitment, dauer execution and dauer exit) is critical for survival in a harsh environment. Finally, one important issue that remains unresolved is the molecular identity of the master regulator protein—the dauer pheromone receptor—that first receives the dauer pheromone signal and subsequently directs worms into either the diapause or normal growth path. The identity of this protein may be uncovered by elucidating the daumone-receptor signaling pathway in its entirety.

## Materials and Methods

### *C. elegans* strains

Strains were maintained as described by Brenner at 20°C unless otherwise specified. *E. coli* OP50 was used as the food source and the wild-type nematodes were the *C. elegans* Bristol variety, strain N2. *C. elegans* mutant strains were obtained from the Caenorhabditis Genetics Center (CGC). Clones stably expressing RNAi (Geneservice, Cambridge, U.K.) against the following genes were obtained for RNAi investigation of FA metabolism during Dauer entry: *acl-2* (T06E8.1), *acl-12* (C01C10.3), *acl-9* (ZK40.1), *acl-5* (R07E3.5), *acl-10* (F55A11.5), *acl-3* (ZK809.2), *acl-1* (F59F4.4), *acl-6* (F08F3.2), *elo-2* (F11E6.5), *fat-1* (Y67H2B.A), *fat-6* (VZK822L.1), *fat-5* (W06D12.3), *fat-2* (W02A2.1), *fat-3* (W08D2.4), *fat-4* (T13F2.1), *fat-7* (F10D2.9), *acs-2* (F28F8.2), *pod-2* (W09B6.1), phospholipase A2, (C03H5.4), *kat-1* (T02G5.8).

### Dauer-inducing assays

Dauer inducing assays were performed as previously described by Jeong *et al*. [3].

### RNA isolation

Worms were stored in RNAlater® (Qiagen, Valencia, CA) for RNA stabilization and were ground under liquid N_2_. Total RNA was extracted with TRIzol reagent (Invitrogen, Carlsbad, CA) and the RNeasy Mini kit® (Qiagen, Valencia, CA). The purity and integrity of total RNA were monitored with NanoDrop® (NanoDrop Technologies) and the Experion® (Biorad), respectively.

### Microarray analysis

Changes in transcript abundance were measured using *C. elegans* whole-genome oligonucleotide microarrays (Affymetrix), replicated in triplicate experiments. All Affymetrix protocols were performed at the Affymetrix Genechip Analysis and Training center of SeouLin Bioscience. cRNA probes were generated using standard Affymetrix protocols (www.affymetrix.com). Fragmented, biotinylated probes were then hybridized to *C. elegans* whole-genome arrays. Washing, labeling (streptavidin-phycoerythrin) and scanning were performed according to standard procedures at the AATC of SeouLin Bioscience.

### Data analysis and statistics

The overall target-specific intensity was obtained by calculating the difference between the intensity of perfect match and mismatch probes. GeneChip Operating Software (Affymetrix, CA) was used to determine the absolute analysis metrics (Detection, Detection p-value) using the scanned probe array data. Metrics were compared between the different treatment-group signals to generate the Change, Change p-value, and Signal Log Ratio (fold change). For normalization, data from each expression array were scaled so that the overall fluorescence intensity across each chip was equivalent (average target intensity set at 500). A one-sided Wilcoxon's signed-rank test was employed to generate the detection p-value. If the overall intensity of a perfect match were much larger than that of mismatch, the detection p-value would be small. The probe set was regarded as present when the p-value was less than 0.05; when the p-value was larger than 0.065, the probe set was regarded as absent. For a given gene transcript in any chip-to-chip comparison, GeneChip Operating Software generates a “change call” parameter (“Induction” or “Suppression”) based on signal specificity as well as intensity. Thus, the change call is based on an evaluation of the intensities of the signals generated from each gene transcript on one chip relative to the corresponding signal intensities on the other chip. Consequently, all “Increase” or “Decrease” calls in comparisons between arrays derived from the same target preparation were defined as false positives. Specification of many gene annotations was also supplemented by further online database searches. Gene expression within each group was categorized into several classes (see also Figure 2B) after clustering using SOM GeneCluster2.0 (http://www.broad.mit.edu/cancer/software/). Gene expression data used for SOM cluster analyses were divided into three groups—a highest confidence set, a high confidence set, and a normal confidence set—according to the p-value obtained in microarray experiments.

### Construction of the dauer database

DauerDB (www.dauerdb.org) was developed by PHP utilizing mySQL. Graphics are supported by the use of GD and gnuplot. The current system is operated by an Apache-based web server. A brief description of DauerDB construction, including GO clustering [48], is as follows: As data is deposited, it is linked to an annotation server, which maintains the updated annotation information for the corresponding gene or signaling pathways. Our system allows a user to access microarray-based genomic expression data derived from an assessment of time-course (life-span)–related gene expression in *C. elegans*. Since the most convenient way to examine differential patterns of gene expression would be to access gene expression information organized according to specific metabolic or signaling pathways, we have integrated the microarray DB into a signaling map using the KEGG pathway to facilitate measurement of relative expression levels under different environmental conditions. For efficient management of DauerDB, we established different levels of access authorization, allowing individual researchers to establish different classes of genes whose accession numbers can be tagged to meet their specific requirements. The microarray database enables Gene Ontology (GO) clustering by extracting GO annotation information from Wormbase (http://www.wormbase.org/), allowing genes to be classified according to function and localization (http://www.geneontology.org/). DauerDB also contains blast2go predictive clustering data, making it possible to cluster genes that lack annotations based on their relative expression levels. DauerDB can also incorporate data produced by the SOM clustering tool, GeneCluster2 [16], which can regroup genes according to the level of gene expression. Through an annotation server, one can get annotation information from *C. elegans* web-based databases. This annotation server incorporates a web robot that retrieves updated data from WormBase, AffyDatabase and KEGG, and uses a script to prepare probe information in XML format.

### qRT-PCR

qRT-PCR was performed using aliquots of the same cDNA samples used for microarray analysis, according to Van Gilst et al. [49]. The primer sequences for all genes were designed using Biotools software at http://biotools.umassmed.edu. The oligonucleotide sequences are available upon request. Relative % expression was determined using the ΔCt method, and an average of the expression of the reference gene, *act-1*, was used to control for template levels. Each experiment was performed in triplicate.

### Fat staining and RNAi analysis

Nile-Red staining of stored fat in worms was performed according to Ashrafi et al. [50]. Sudan-black staining of fat storage in worms that had become dauers was performed using worms fixed with 1% paraformaldehyde, washed, dehydrated with ethanol and stained with dye [3]. RNAi analysis of fat-related genes was performed according to published procedures [51]. Briefly, bacteria containing each RNAi clone (Geneservice, Cambridge, U.K.) were cultured for 6–14 h in 5 ml Luria Broth media containing 50 µg/ml ampicillin. A 40-µl aliquot of each culture was spotted onto a petri dish containing NGM agar and 1 mM IPTG. After overnight incubation, Nile Red was added on top of each well to a final concentration of 0.05 µg/ml. Approximately 100 eggs were placed in each well and incubated at 25°C. Growth and Nile-Red staining were assessed after 22 h by light phase and UV microscopy. For each batch of RNAi clones tested, L4440 (vector alone) and OP50 control wells were included. All phenotypes were confirmed by at least two additional rounds of testing on the selected clones.

### Fluorescence microscopy, image acquisition and intensity quantitation

Nile-Red fluorescence was visualized using a Zeiss AXIO microscope equipped with rhodamine (emission 560–590 nm) filters. Images were captured using a digital AXIOcam HRc attached to a Zeiss Axioplan II microscope equipped with a rhodamine filter. All Nile-Red images were acquired using identical settings and exposure times (100 and 200 ms). To quantify pixel intensities and numbers, equivalent planes and regions of the worm body were selected.

## Supporting Information

Figure S1 Structure and detailed description of dauer database (A) List page of microarray database. (B) The distribution map of genes annotated to microtubule motor activity (GO∶3777). The number to the right of the Gene Ontology term indicates total gene number annotated with this Gene Ontology term (i.e., 11349). In the gene ontology list, “[dist]” is the distribution map link of total genes annotated to the GO term. The distribution map has two parts - stage ratio distribution and stage distribution; the former shows the expression ratio between stages, the latter displays the distribution of gene expression lists. (C) Clusters were regrouped based on manual annotation and the expression maps of each cluster. For SOM clustering, 10×10 or 8×8 dimension parameters were used. In cluster titles, “_sub” cluster is regrouped by manual annotation after SOM clustering. (D) Glycolysis pathway from KEGG map. The green box is the position of the enzyme in the *C. elegans* metabolic pathway. (E) Detailed expression information for a single gene.
(0.66 MB DOC)

Figure S2 The global assessment of gene expression during entry of *C. elegans* into the dauer state. SOM clustering of the resulting expression profiles of worms grown on daumone plates (S1, S2, S3, S4, S5, S6) and NGM plates without daumone (L1, L2, L3), and in liquid culture in which the latter were induced to form dauer larvae (Da) by culturing for >360 h. Total RNA was prepared as described in “Experimental Procedures”. The expression value of each gene is depicted as a color gradient according to the relative ratio of the detection value. White represents the highest level of expression (induction); black, the lowest level of expression (suppression) during dauer entry. Triplicate samples were analyzed at each stage; each column represents one set of experiments. At the right- and left-hand sides, eight (Path 1) and six (Path 2) distinct expression profile patterns from each experimental condition are grouped using the SOM clustering method to depict changes in gene expression under different growth condition.
(0.35 MB DOC)

Figure S3 Quantitative RT-PCR for selected aging-related genes involved in the insulin signaling pathway. Shown here are qRT-PCR data of ten representative genes from the aging- and oxidative stress-related signaling pathways, as depicted in Figure 3. On the left is microarray data and on the right is qRT-PCR data, which show patterns consistent with the microarray data.
(0.17 MB DOC)

Figure S4 Quantitative RT-PCR of genes involved in fatty acid metabolism. Each cDNA sample for microarray was analyzed by qRT-PCR. cDNA quality and quantity were determined using NanoDrop® (NanoDrop Technologies). PCR was performed using the SYBR Green PCR Master Mix (Qiagen) according to the manufacturer's instructions, and reactions were run on a DNA Engine Opticon® 2 System (MJ Research). Primer sequences for all genes were designed using Biotools software (http://biotools.umassmed.edu/). Relative % expression was determined using the ΔC**t** method, and an average of the expression of the reference gene, *act-1*, was used to control for template levels. Each experiment was performed in triplicate.
(0.41 MB DOC)

Figure S5 Changes in the expression of genes associated with specific metabolic pathways during dauer entry on daumone plates (Path 1). Differential expression of genes associated with various metabolic pathways and energy production during dauer entry. The genes selected in each cluster are based on GO clustering and sequence homology. The histogram in each column represents the relative level of expression; the right column shows the numbers of genes detected in Path 1.
(0.41 MB DOC)
