# Correction: Molecular Time-Course and the Metabolic Basis of Entry into Dauer in *Caenorhabditis elegans*

**DOI:** 10.1371/annotation/0dfbcb98-872c-4e20-96e0-5deb7f484830

**Published:** 2009-02-03

**Authors:** Pan-Young Jeong, Min-Seok Kwon, Hyoe-Jin Joo, Young-Ki Paik

Figure S4 is a duplicate of Figure S5. Please view the correct Figure S4 here: 
