# Supplementary figures and images for: Molecular Time-Course and the Metabolic Basis of Entry into Dauer in Caenorhabditis elegans
Source: PLoS One. 2009 Jan 8;4(1):e4162. doi: 10.1371/journal.pone.0004162 (PMC2612749; doi:10.1371/journal.pone.0004162)

Figure S1A


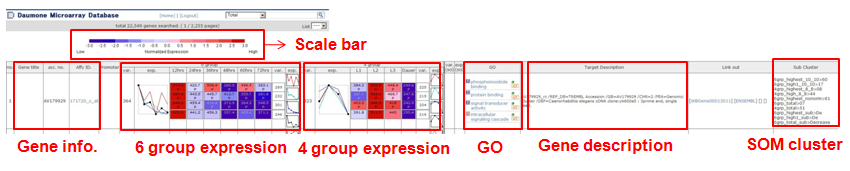


Figure S1B


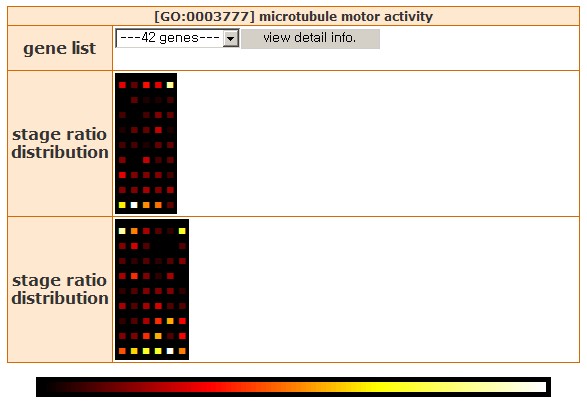


Figure S1C


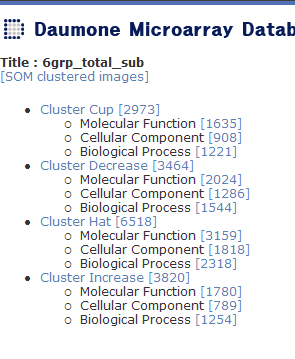

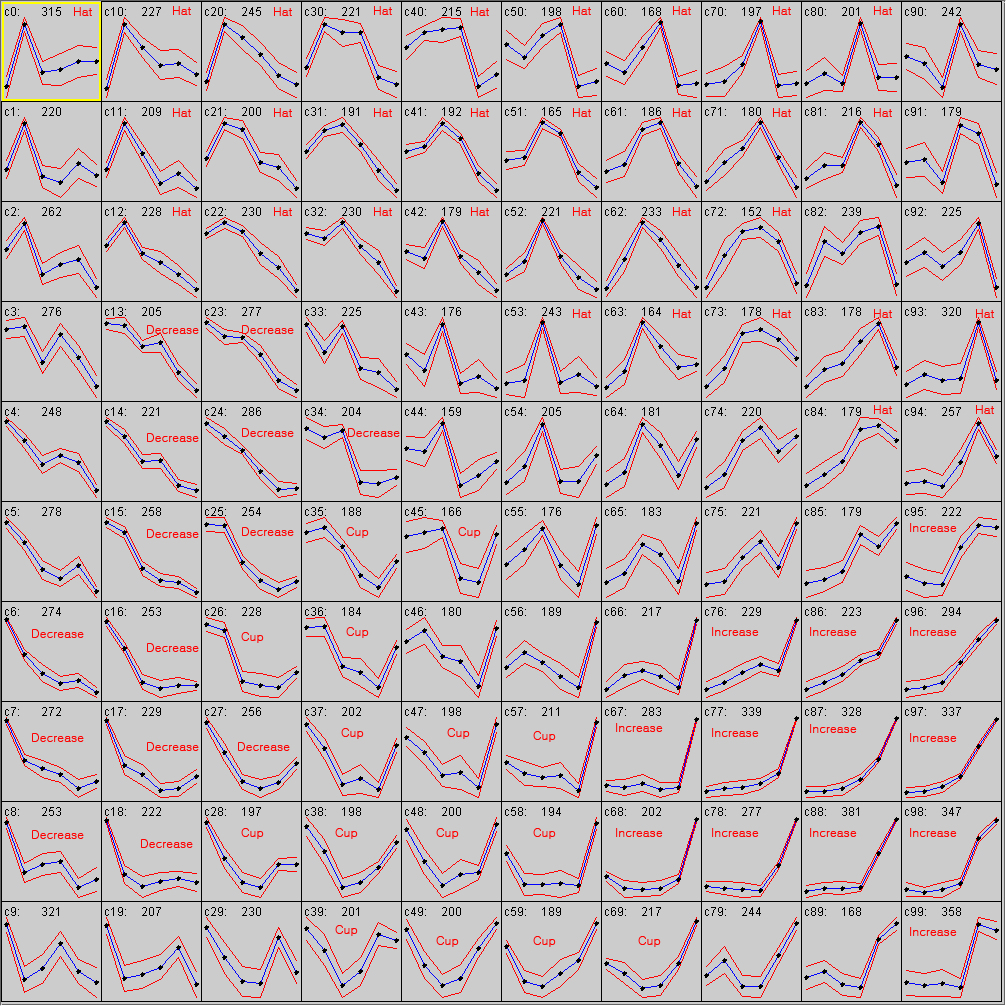


Figure S1D


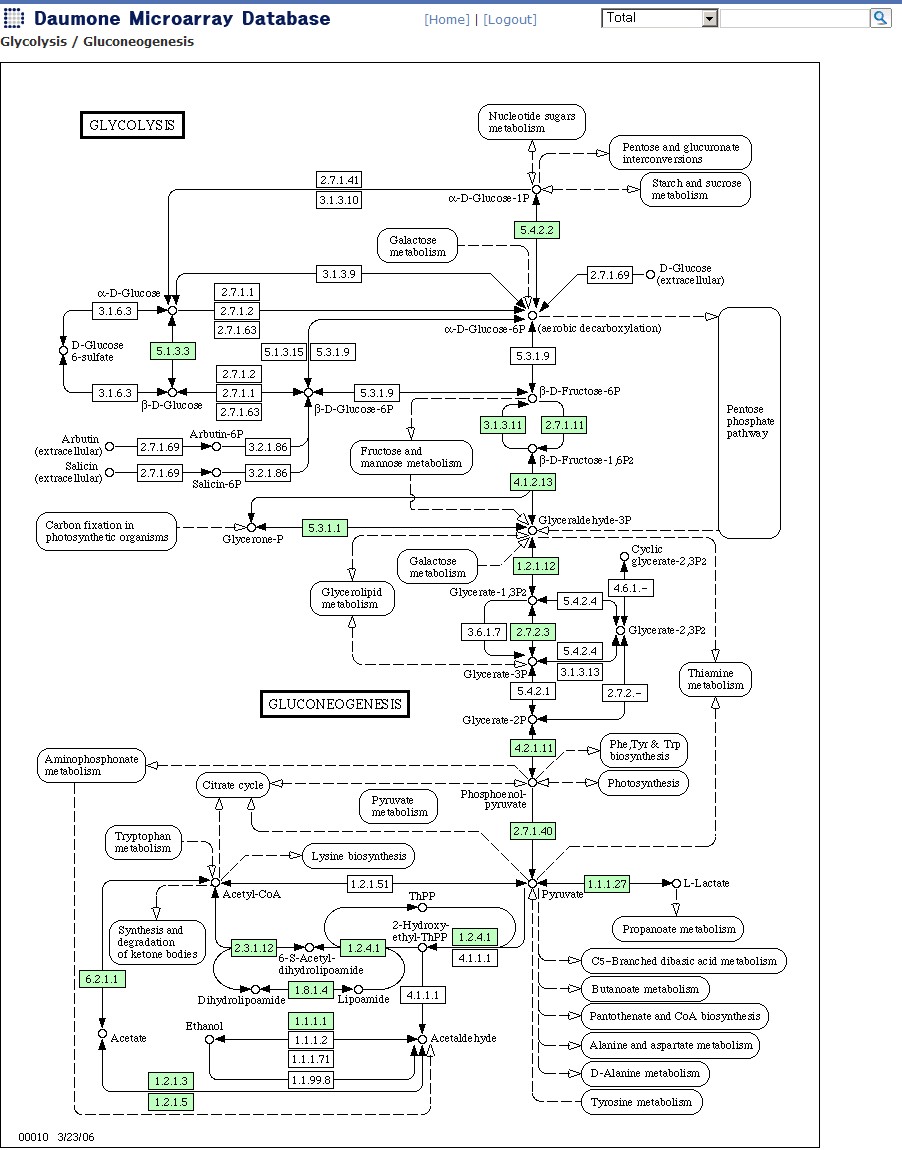


Figure S1E


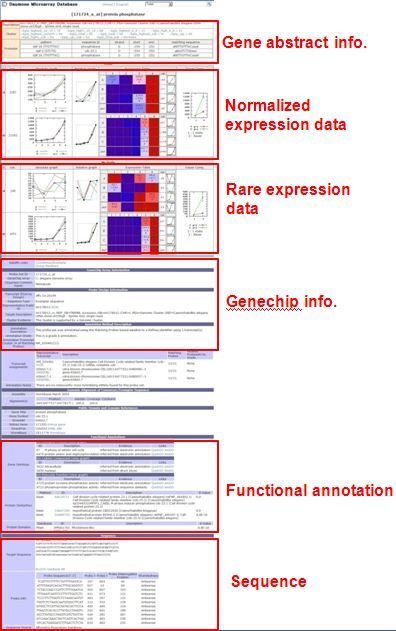

Supplement: Figure S1 — Structure and detailed description of dauer database (A) List page of microarray database. (B) The distribution map of genes annotated to microtubule motor activity (GO∶3777). The number to the right of the Gene Ontology term indicates total gene number annotated with this Gene Ontology term (i.e., 11349). In the gene ontology list, “[dist]” is the distribution map link of total genes annotated to the GO term. The distribution map has two parts - stage ratio distribution and stage distribution; the former shows the expression ratio between stages, the latter displays the distribution of gene expression lists. (C) Clusters were regrouped based on manual annotation and the expression maps of each cluster. For SOM clustering, 10×10 or 8×8 dimension parameters were used. In cluster titles, “_sub” cluster is regrouped by manual annotation after SOM clustering. (D) Glycolysis pathway from KEGG map. The green box is the position of the enzyme in the C. elegans metabolic pathway. (E) Detailed expression information for a single gene. (0.66 MB DOC) [file pone.0004162.s001.doc]

Figure S2


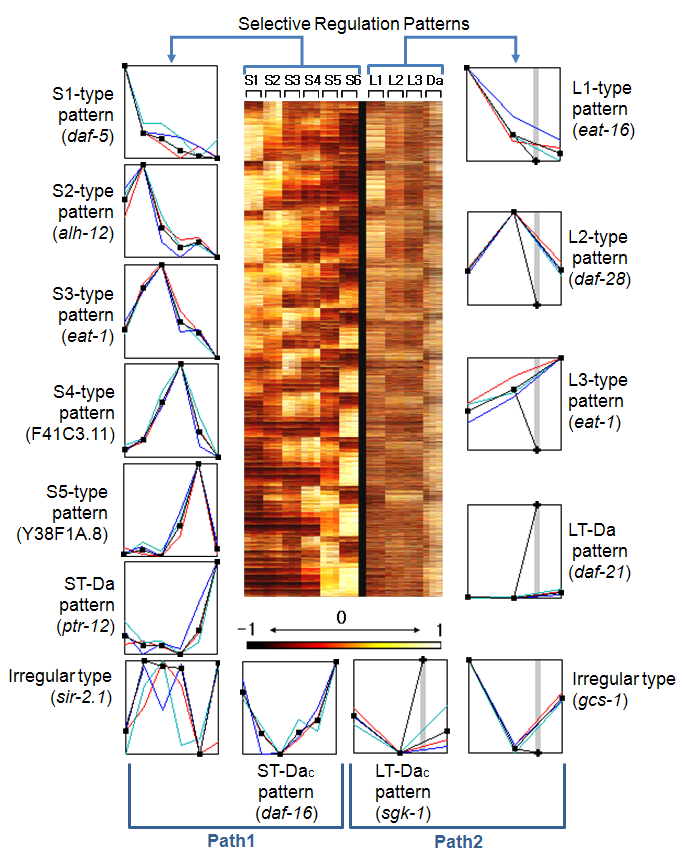

Supplement: Figure S2 — The global assessment of gene expression during entry of C. elegans into the dauer state. SOM clustering of the resulting expression profiles of worms grown on daumone plates (S1, S2, S3, S4, S5, S6) and NGM plates without daumone (L1, L2, L3), and in liquid culture in which the latter were induced to form dauer larvae (Da) by culturing for >360 h. Total RNA was prepared as described in “Experimental Procedures”. The expression value of each gene is depicted as a color gradient according to the relative ratio of the detection value. White represents the highest level of expression (induction); black, the lowest level of expression (suppression) during dauer entry. Triplicate samples were analyzed at each stage; each column represents one set of experiments. At the right- and left-hand sides, eight (Path 1) and six (Path 2) distinct expression profile patterns from each experimental condition are grouped using the SOM clustering method to depict changes in gene expression under different growth condition. (0.35 MB DOC) [file pone.0004162.s002.doc]

Figure S3

**
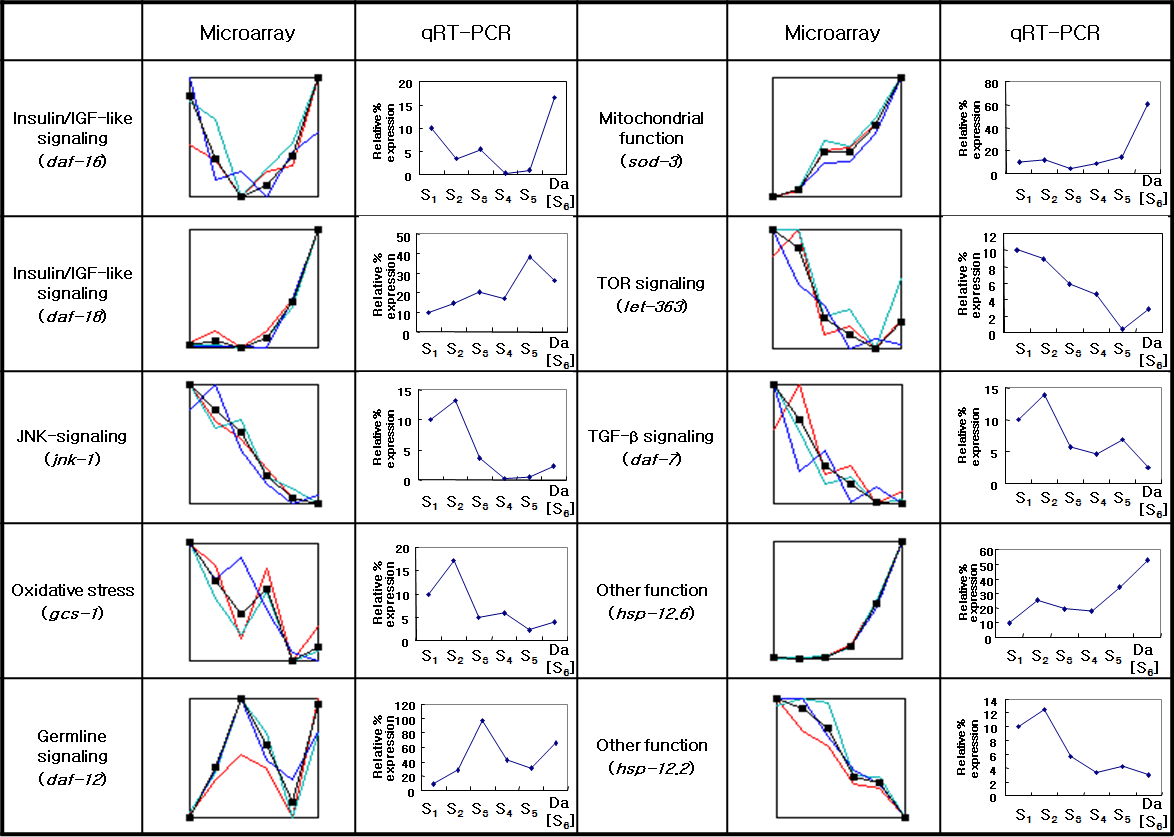
**

Supplement: Figure S3 — Quantitative RT-PCR for selected aging-related genes involved in the insulin signaling pathway. Shown here are qRT-PCR data of ten representative genes from the aging- and oxidative stress-related signaling pathways, as depicted in Figure 3. On the left is microarray data and on the right is qRT-PCR data, which show patterns consistent with the microarray data. (0.17 MB DOC) [file pone.0004162.s003.doc]

Figure S4

**
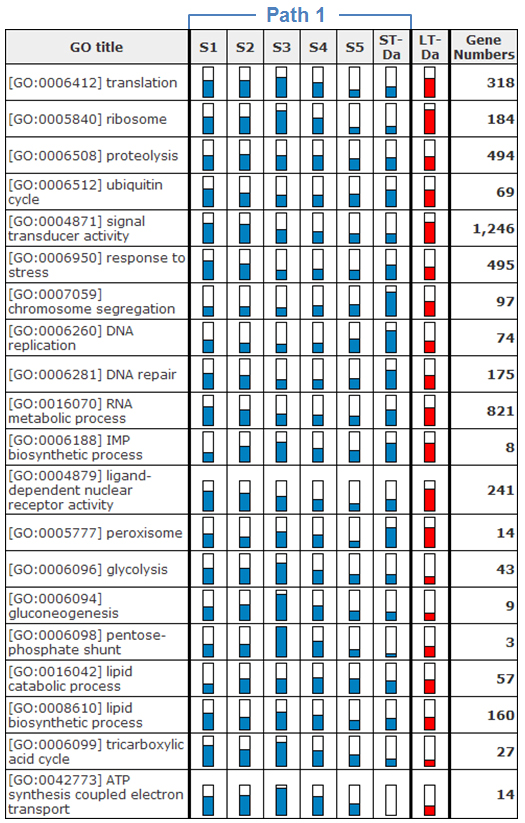
**

Supplement: Figure S4 — Quantitative RT-PCR of genes involved in fatty acid metabolism. Each cDNA sample for microarray was analyzed by qRT-PCR. cDNA quality and quantity were determined using NanoDrop® (NanoDrop Technologies). PCR was performed using the SYBR Green PCR Master Mix (Qiagen) according to the manufacturer's instructions, and reactions were run on a DNA Engine Opticon® 2 System (MJ Research). Primer sequences for all genes were designed using Biotools software (http://biotools.umassmed.edu/). Relative % expression was determined using the ΔCt method, and an average of the expression of the reference gene, act-1, was used to control for template levels. Each experiment was performed in triplicate. (0.41 MB DOC) [file pone.0004162.s004.doc]

Figure S5

**
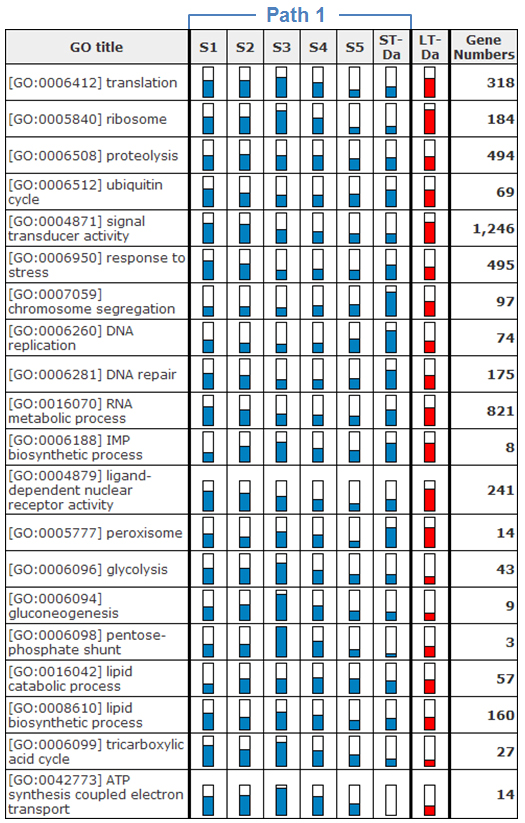
**

Supplement: Figure S5 — Changes in the expression of genes associated with specific metabolic pathways during dauer entry on daumone plates (Path 1). Differential expression of genes associated with various metabolic pathways and energy production during dauer entry. The genes selected in each cluster are based on GO clustering and sequence homology. The histogram in each column represents the relative level of expression; the right column shows the numbers of genes detected in Path 1. (0.41 MB DOC) [file pone.0004162.s005.doc]
