# Supplementary figures and images for: Correction: Molecular Time-Course and the Metabolic Basis of Entry into Dauer in Caenorhabditis elegans
Source: PLoS One. 2009 Feb 3;4(2):10.1371/annotation/0dfbcb98-872c-4e20-96e0-5deb7f484830. doi: 10.1371/annotation/0dfbcb98-872c-4e20-96e0-5deb7f484830 (PMC2646300; doi:10.1371/annotation/0dfbcb98-872c-4e20-96e0-5deb7f484830)

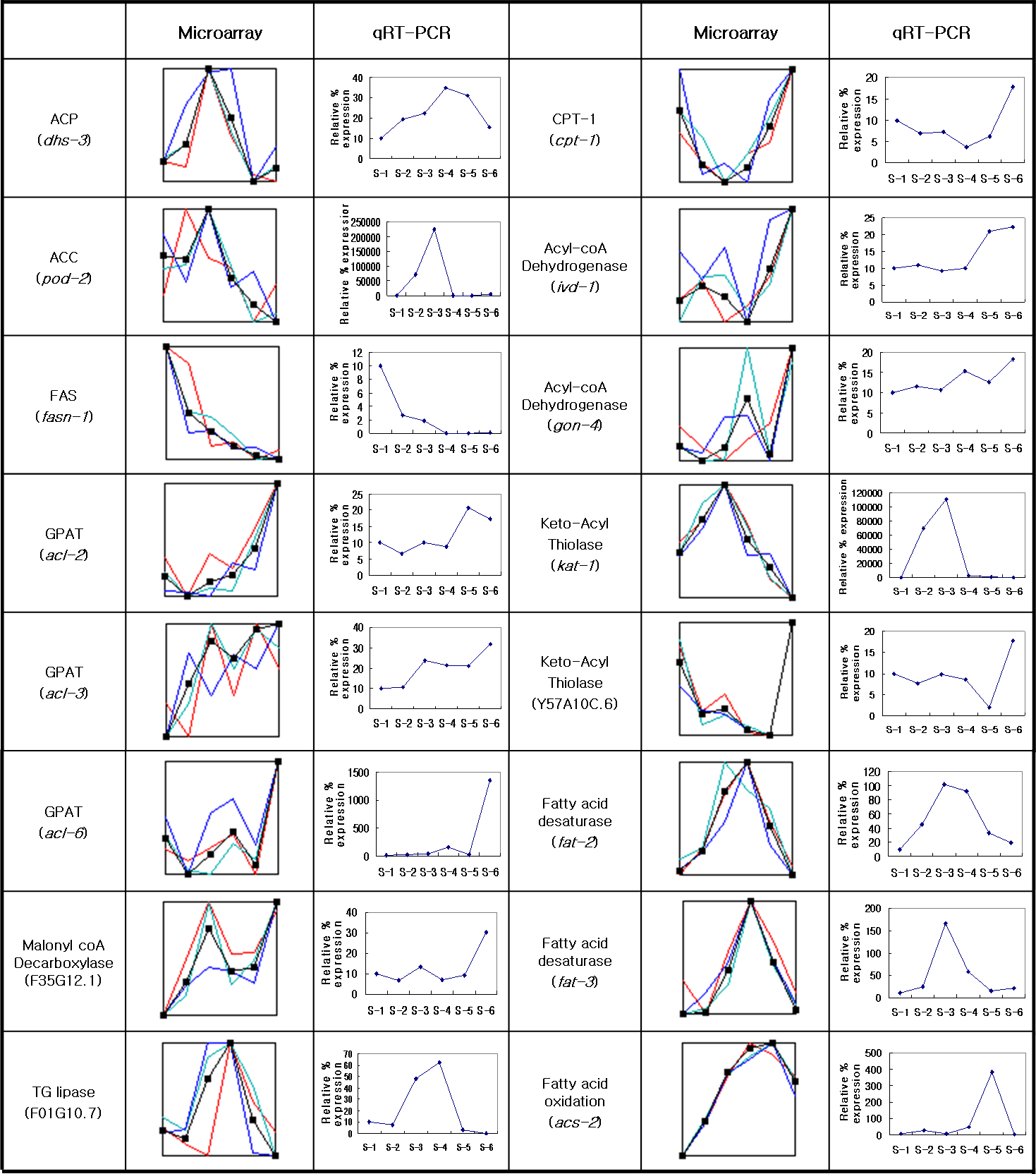

Supplement: Supplementary file 1 [file pone.0dfbcb98-872c-4e20-96e0-5deb7f484830.s001.tif]
